# Role of Operon *aao_So_-mutT* in Antioxidant Defense in *Streptococcus oligofermentans*

**DOI:** 10.1371/journal.pone.0038133

**Published:** 2012-05-30

**Authors:** Peng Zhou, Lei Liu, Huichun Tong, Xiuzhu Dong

**Affiliations:** State Key Laboratory of Microbial Resources, Institute of Microbiology, Chinese Academy of Sciences, Beijing, China; The Methodist Hospital Research Institute, United States of America

## Abstract

Previously, we have found that an insertional inactivation of *aao_So_*, a gene encoding L-amino acid oxidase (LAAO), causes marked repression of the growth of *Streptococcus oligofermentans*. Here, we found that *aao_So_* and *mutT*, a homolog of pyrophosphohydrolase gene of *Escherichia coli*, constituted an operon. Deletion of either gene did not impair the growth of *S. oligofermentans*, but double deletion of both *aao_So_* and *mutT* was lethal. Quantitative PCR showed that the transcript abundance of *mutT* was reduced for 13-fold in the *aao_So_* insertional mutant, indicating that gene polarity derived from the inactivation of *aao_So_* attenuated the expression of *mutT*. Enzymatic assays were conducted to determine the biochemical functions of LAAO and MutT of *S. oligofermentans*. The results indicated that LAAO functioned as an aminoacetone oxidase [47.75 nmol H_2_O_2_ (min·mg protein)^–1^]; and MutT showed the pyrophosphohydrolase activity, which removed mutagens such as 8-oxo-dGTP. Like paraquat, *aao_So_* mutations increased the expression of SOD, and addition of aminoacetone (final concentration, 5 mM) decreased the mutant’s growth by 11%, indicating that the *aao_So_* mutants are under ROS stress. HPLC did reveal elevated levels of cytoplasmic aminoacetone in both the deletion and insertional gene mutants of *aao_So_*. Electron spin resonance spectroscopy showed increased hydroxyl radicals in both types of *aao_So_* mutant. This demonstrated that inactivation of *aao_So_* caused the elevation of the prooxidant aminoacetone, resulting the cellular ROS stress. Our study indicates that the presence of both LAAO and MutT can prevent endogenous metabolites-generated ROS and mutagens. In this way, we were able to determine the role of the *aao_So_-mutT* operon in antioxidant defense in *S. oligofermentans*.

## Introduction

Oxidative stress is encountered universally by all organisms living in an oxygen environment. Oxygen is a relatively inert molecule in nature. However, when it is channeled to electron- transfer reactions, especially inside the cells that experience auto-oxidation of reduced enzyme cofactors, oxygen is converted into deleterious reactive oxygen species (ROS). Due to the higher energy that results from the presence of unpaired valence-shell electrons, ROS can cause severe damage to various biological macromolecules, especially DNA. This leads to mutagenesis, tumorigenesis, and aging [1], [2].

Organisms have developed various means of resisting oxidative stress. Aerobes generally employ superoxide dismutase (SOD) and catalase cascades to eliminate cellular ROS. SOD dismutes superoxide anions as H_2_O_2_, which is then converted to H_2_O and O_2_ by catalase [3], [4]. Anaerobes use the superoxide reductase (SOR)-dependent oxide decomposition pathway [5]. SOR reduces superoxide to H_2_O_2_ using reduced rubredoxin (Rd_red_) as the electron donor. Then, in concert with the non-heme peroxidase rubrerythrin, SOR reduces H_2_O_2_ to water. Intact H_2_O_2_ is not particularly deleterious to cells, but H_2_O_2_-derived hydroxyl radicals are indiscriminately damaging to the biological macromolecules. The conversion of H_2_O_2_ into radicals takes place through Fenton chemistry, which is activated by ferrous or copper ions [6]–[8]. Streptococci, which are facultative anaerobes, undergo fermentative metabolism to produce energy reserves. Streptococci do not possess catalase; instead, they have SOD and other oxidases. The formation of H_2_O_2_ is their main means of attenuating ROS damage [4], [6]. *Streptococcus oligofermentans* is isolated from non-caries dental plaques in humans. It uses multiple pathways for the generation of H_2_O_2_ from oxygen [9]. These pathways include lactate oxidase (Lox) in oxidation of lactate to produce H_2_O_2_ [10]; L-amino acid oxidase (LAAO) to oxidize amino acids to H_2_O_2_ [11]; and a recently identified pyruvate oxidase (Pox) [12]. Lox and Pox contribute the majority of the H_2_O_2_ derived from glycolysis products [12], and LAAO generates lower levels of H_2_O_2_ when proteinaceous substances are present. Among streptococci, *S. oligofermentans* is one of those with the highest levels of tolerance to H_2_O_2_ [13]. This makes it a suitable model organism for the study of the mechanisms by which bacteria protect themselves against oxidative stress.

In a previous study on LAAO, we found that insertional inactivation of *aao_So_* caused severe reductions in the growth of *S. oligofermentans* [11]. To gain insight into the growth retardation, we performed a microarray assay and observed the differential expression of some genes in *aao_So_* mutants, including the genes encoding manganese-dependent SOD (*sodA*), DNA-binding ferritin-like protein, DNA repair enzyme, and NADH oxidase II (*nox*). Elevated H_2_O_2_ yield was detected in glucose-containing cultures of *aao_So_* mutants. These results imply that inactivation of *aao_So_* causes an elevation of cellular ROS levels in *S. oligofermentans*.

In the present study, we used genetic and biochemical analyses to find that LAAO is an authentic aminoacetone oxidase. It reduces the aminoacetone-derived ROS *in vivo*. Furthermore, *aao_So_*, by constituting an operon with *mutT*, encoding a nucleotide-oxide scavenging protein, develops a dual guard by reducing the production of cellular ROS and clearing the nucleotide oxides generated by ROS in streptococci. Thus, a novel antioxidant defense gene operon has been demonstrated.

## Results

### Identification of the *aao_So_-mutT* Operon in *S. oligofermentans*

**Figure 1:**
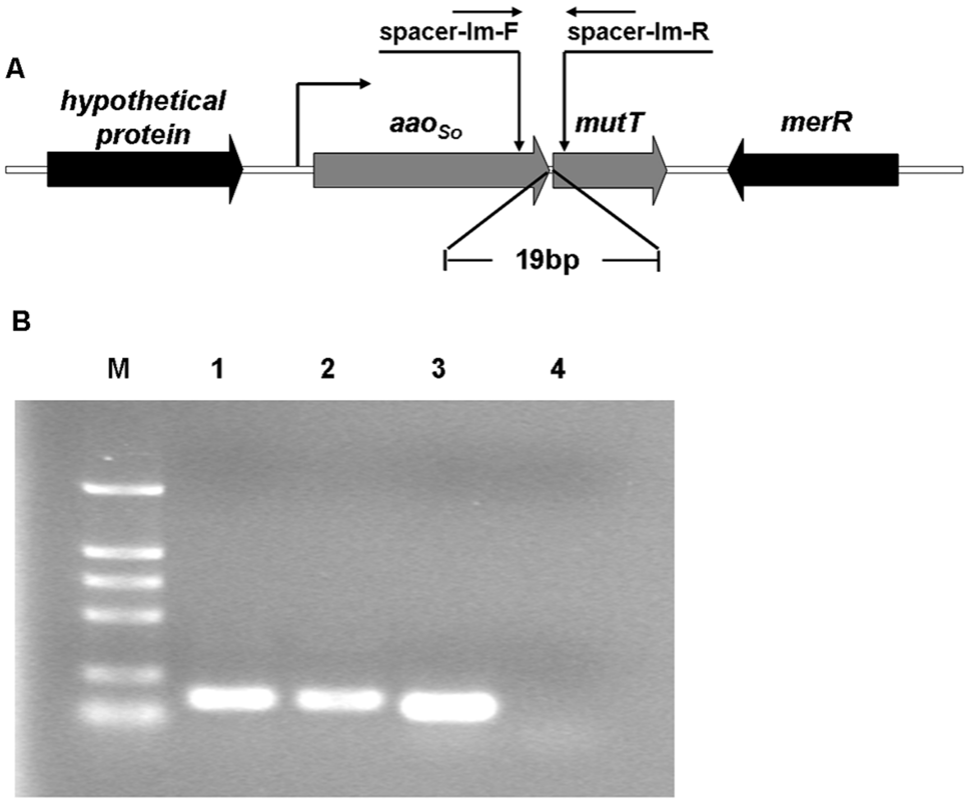
Cotranscription of *aao_So_* and *mutT* in *S. oligofermentans*. (A) Schematic arrangement of *aao_So_* and *mutT* in the genome of *S. oligofermentans.* (B) Agarose gel electrophoresis of the RT-PCR products for the intergenic spacer between *aao_So_* and *mutT*. M, DL2000 marker; 1, PCR products amplified with primer spacer-lm-F and spacer-lm-R with gDNA as template; 2, PCR products amplified with primer spacer-lm-F and spacer-lm-R using cDNA as template; 3, PCR products amplified with primer spacer-neg-F and spacer-neg-R with gDNA as template; 4, PCR products amplified with primer spacer-neg-F and spacer-neg-R with cDNA as template.

Previous studies have found that an insertional mutation of *aao_So_* decreased the growth of *S. oligofermentans* to two thirds that of the wild-type strain [11]. This suggests that other inhibitory mechanisms might be present in addition to the H_2_O_2_ generated by LAAO. To evaluate possible inhibitive causes, we examined the genes that flanked *aao_So_* in the genome of *S. oligofermentans*. As shown in Fig. 1A, a putative gene encoding MutT (Acc No. JQ083601), a homolog of pyrophosphohydrolase (8-oxo-dGTPase) in *Escherichia coli* [14], was located 19 bp downstream of *aao_So_*. The two appear to constitute an operon. Then, using a reverse transcription-PCR assay, we determined that *aao_So_* and *mutT* were cotranscribed (Fig. 1B). This confirmed the presence of the operon and coexpression of the two genes in *S. oligofermentans*.

### Role of Coexistence of *aao_So_* and *mutT* in *S. oligofermentans*

**Figure 2:**
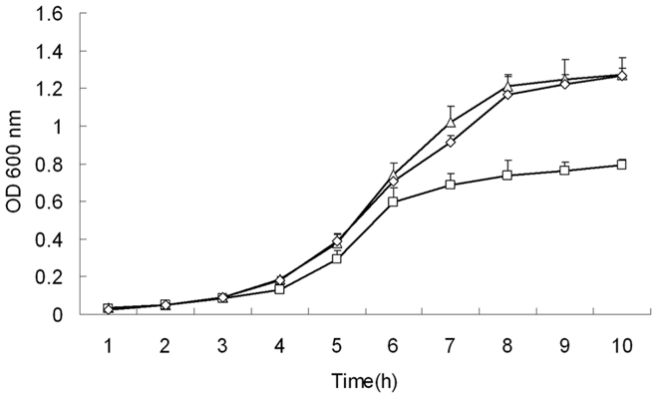
Growth of the wild-type, *Δaao_So_-mutT* mutant of *S. oligofermentans* and its complementary strain in glucose. Strains were pre-cultured overnight in TYG medium, and 1∶100 diluted with fresh TYG medium with the same OD_600_. Samples were taken every 1 h to detect OD_600_. Symbols: △, wild-type strain; □, *Δaao_So_-mutT* mutant; ◊, *Δaao_So_-mutT* complementary strain. The results are shown as the means±SD of three independent experiments.

To determine the relative effects of *aao_So_* and *mutT* on the growth of *S. oligofermentans*, we constructed deletion mutants for each gene. As shown in Fig. S1, the deletion mutant *Δaao_So_* exhibited slightly slower growth than wild-type strain, and?*ΔmutT* did not show any detectable decrease in growth. A complementary strain of the insertional mutant of *aao_So_* resumed growth (Fig. 2). However, we never obtained a double gene mutant, here denoted *Δaao_So_-ΔmutT*, even under obligate anaerobic conditions by manipulating inside an anaerobic chamber. This implies that double mutation of the two genes may be lethal. To confirm this, we first cloned the *aao_So_-mutT* operon into the *E. coli*-*streptococcus* shuttle-expression vector pDL278 and then introduced it into the wild-type strain of *S. oligofermentans*. Under this background, we readily deleted *aao_So_-mutT* from the chromosome. This mutant also carried a plasmid containing the operon, which showed growth patterns similar to the wild-type strain. To clarify the effect of an insertional mutation of *aao_So_* on the downstream *mutT*, we determined the expression of *mutT* by quantitative PCR and found a 13-fold reduction in transcript abundance relative to that of the wild-type strain, but in the *Δaao_So_* strain, the deletion mutant, *mutT* expression was similar to the wild-type (Table 1). This indicates that *aao_So_* inactivation caused a gene-polarity effect on *mutT*. This insertional mutant was designated *Δaao_So_-mutT*, and the suppression of growth was predicted to be related to the loss of MutT activity. Taken together, the coexistence of *aao_So_* and *mutT* seems to be essential for *S. oligofermentans* to carry out its normal physiological functions and even survive.

**Table 1:** Transcript levels of *mutT* and *mutX* in the wild-type, *Δaao_So_* and *Δaao_So_-mutT* mutants of *S. oligofermentans*.

	The number of gene transcript*	
Strains	*mutt*	*mutX*
wild-type	0.66±0.15	0.03±0.01
*Δaao_So_-mutT*	0.05±0.01	ND
*Δaao_So_*	0.76±0.17	ND

* The transcript copies are given as the means±SD/10,000 16S rRNA copies.

ND, not determined.

### LAAO Authentically Acts as an Aminoacetone Oxidase

Enzymatic activity was assayed to clarify the function of LAAO. Our previous study showed that LAAO, an L-amino acid oxidase, mainly oxidizes seven kinds of amino acids to generate H_2_O_2_ [11]. Aminoacetone belongs to the amine family, and is determined as an electron donor in reactions involving active transition metals. So that it generates superoxide anions (ROS) from oxygen [15]. In human cells, semicarbazide-sensitive amine oxidase (SSAO) has been shown to remove aminoacetone by converting it to methylglyoxal (MG) and H_2_O_2_ [15], [16]. Given that aminoacetone is an analog of amino acids, we tested the enzymatic activity of the overexpressed LAAO on aminoacetone. By using 25 mM of aminoacetone as substrate and air as an oxygen supply, we detected the production of 47.75 nmol H_2_O_2_ (min·mg protein)^–1^. In parallel, the activity of LAAO on lysine was only 2.22 nmol H_2_O_2_ (min·mg protein)^–1^. Even when lysine concentration was increased to 100 mM, the activity was only 12.29 nmol H_2_O_2_ (min·mg protein)^–1^. Similar low activities were detected for aspartic acid and glutamine (Fig. S2). This indicates that aminoacetone, not amino acids, is the preferred substrate of LAAO. However, unlike the described amine oxidases [17], LAAO did not catalyze benzylamine or methylamine conversion (data not shown).

### Role of MutT in Elimination of Nucleotide Oxides in *S. oligofermentans*

Previous research has shown that dGTP can be readily oxidized to 8-oxo-dGTP by hydroxyl radicals, owing to its hypersensitivity to oxidation [18], [19]. MutT of *E. coli* and its human homolog hMTH-1 protein hydrolyze 8-oxo-dGTP to pyrophosphate (PPi) and the harmless monophosphate 8-oxo-dGMP through a specific pyrophosphohydrolase (8-oxo-dGTPase) activity. Two *mutT* homologs, *mutT* and *mutX*, were found in the genome of *S. oligofermentans.* By using quantitative PCR, we determined that the transcript abundance of *mutT* was about 20-fold higher than that of *mutX* (Table 1), similar to the results of our previous microarray assays (unpublished data).

Next, we determined the pyrophosphohydrolase activity of the purified MutT protein of *S. oligofermentans in vitro*. According to the activity of MutT of *E. coli*, synthetic 8-oxo-dGTP was used as the substrate in the enzymatic assay. Upon addition of the purified recombinant MutT protein from *S. oligofermentans*, reduction of 8-oxo-dGTP levels was detected in the reaction mixture by HPLC (Fig. S3). This confirmed the pyrophosphohydrolase activity of *S. oligofermentans* MutT.

### Effects of Inactivation of *aao_So_* on the Regulation of *sodA*

Our previous microarray data identified increased mRNA from *sodA*, the only SOD-encoding gene in the *S. oligofermentans* genome, by the *aao_So_* mutant relative to the wild-type strain (unpublished data). As superoxide anion is the well-defined substrate of SOD, a link between LAAO protein and the cellular ROS level may exist. To test this hypothesis, quantitative PCR was used to assay the expression of *sodA* in the *aao_So_* mutants of *S. oligofermentans*. As expected, higher transcript abundance of *sodA* was detected in *Δaao_So_-mutT* mutant (76.63±13.62 copies) and *Δaao_So_* mutant (47.94±8.83 copies), relative to the wild-type strain (13.11±3.02 copies). In addition, dramatic increases in Mn-SOD activity were observed in the early and middle log phase cultures of both mutants (Table 2). As *sodA* mRNA levels increase 3-fold in wild-type *S. oligofermentans* treated with the redox-cycling drug paraquat, which induces production of cellular endogenous superoxides (data not shown), we propose that the accumulation of superoxide anion increases the expression of Mn-SOD in *Δaao_So_-mutT* and *Δaao_So_* mutants.

**Table 2:** Mn-SOD activities of deletion and insertion mutants of *S. oligofermentans aao_So_*.

Specific activity (U mg^−1^ protein)*			
Growth time	wild-type	*Δaao_So_*	*Δaao_So_-mutT*
4 h	2.55±0.68	29.38±9.49	34.89±4.92
6 h	17.88±5.33	43.43±10.54	45.62±8.68

* Activity was detected using the method of pyrogallol auto-oxidation [40]. Data were obtained as means±SD from three independent experiments.

We then constructed *ΔsodA* and *Δaao_So_-mutT-ΔsodA* mutants to confirm the superoxide-induced impairment of growth. As expected, no obvious decrease in growth was observed for *ΔsodA* mutant. However, a reduction in growth of nearly 70% was detected for the double mutant, *Δaao_So_-mutT-ΔsodA* (Fig. S4). Whereas when grew under strictly anaerobic conditions, *Δaao_So_-mutT* mutant grew to a final OD_600_ of 1.2, similar to wild-type; and the growth of the mutant *Δaao_So_-mutT-ΔsodA* was restored to a level similar to that of the insertional mutant of *aao_So_*. This implies that superoxide anions may be the key to impairing the growth of *Δaao_So_-mutT* mutants.

### Effects of Mutation of *aao_So_* on Cellular Levels of ROS

**Figure 3:**
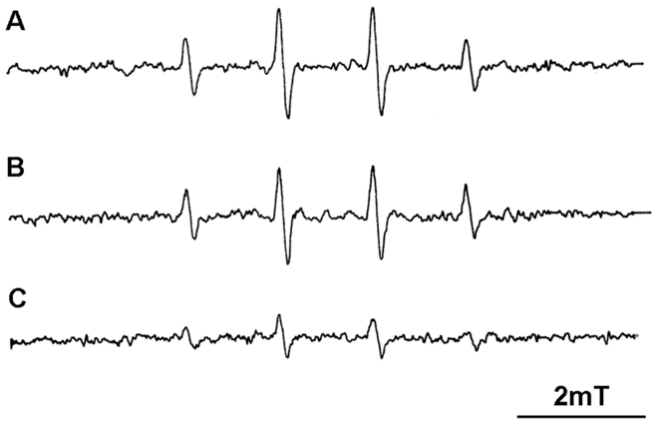
ESR spectra of the DMPO treated cells of *S. oligofermentans.* The characteristic 1∶2:2∶1 quartet of hydroxyl radical adduct of DMPO signals were shown in the DMPO-contained cell suspensions of (A) *Δaao_So_-mutT* mutant, (B) *Δaao_So_* mutant, and (C) wild-type strain.

To further evaluate the link between intracellular levels of ROS to LAAO, we measured the ROS levels in the *Δaao_So_-mutT*, *Δaao_So_* mutants and the wild-type strain. Considering that the accumulation of superoxide in cells is concomitant with abundant H_2_O_2_ generated by lactate oxidase and pyruvate oxidase, and either superoxide anion or H_2_O_2_ can cause the release of iron from proteins containing [4Fe–4S]^2+^ clusters, the Fenton reaction can cause an elevation in cellular hydroxyl radicals [20]. Therefore, hydroxyl radicals were assessed in *S. oligofermentans* strains using electron spin resonance (ESR) with 5,5-dimethyl-1-pyrroline-*N*-oxide (DMPO) spin trap [21], [22]. ESR spectroscopy is the most efficient and non-destructive technique for detecting chemical species that have unpaired electrons, such as the superoxide anion radical and hydroxyl radical. As a spin trap, DMPO has been frequently used to detect oxygen-centered radicals [22]. As shown in Fig. 3, intensive and characteristic 1∶2:2∶1 quartet of DMPO-OH^·-^ adduct signal was obtained both in the *Δaao_So_-mutT* and *Δaao_So_* mutants, but only a very weak signal was detected in the wild-type strain. This demonstrates that the *aao_So_* mutants were under a relatively high ROS stress.

### Effects of Mutation of *aao_So_* on Levels of Cytoplasmic Aminoacetone

To evaluate the link between intracellular ROS and the inactivation of LAAO, based on the aminoacetone oxidase activity of LAAO, we assessed the intracellular accumulation of aminoacetone for the wild-type strain, *Δaao_So_-mutT*, and *Δaao_So_* mutants. However, cytoplasmic aminoacetone was not detected in any strain, probably due to the instability of this compound. Using a method described by Kazachkov and Yu, we generated a stable derivative of aminoacetone by addition of 9-fluorenylmethyl chloroformate-Cl (FMOC-Cl) to the cellular lysate [23]. As shown in Fig. S5, levels of aminoacetone as much as double that observed in wild-type (76.8±12.5 pmol mg^−1^ cell) were determined in *Δaao_So_-mutT* mutant (159.8±9.2 pmol mg^−1^ cell) and *Δaao_So_* strain (147.2±21.4 pmol mg^−1^ cell). Similarly elevated levels were observed in semicarbazide-sensitive amine oxidase (SSAO)-repressed human tissues [23]. This determines that LAAO has a function similar to that of amine oxidase in removing the endogenous aminoacetone.

Next, we added 5 mmol aminoacetone to cultures of wild-type, *Δaao_So_-mutT* mutant, and *Δaao_So_* mutant. It was found that aminoacetone reduced the growth of *Δaao_So_-mutT* mutant by 11%, but it increased the growth of the wild-type and *Δaao_So_* mutant by 10% (Table 3). Cellular aminoacetone can be derived from threonine or glycine [24], so we supplied threonine to the mutant cultures. Effects of threonine on growth were similar to those of aminoacetone (data not shown). To confirm that the accumulation of threonine-derived aminoacetone reduces the growth of *S. oligofermentans*, we first constructed a threonine dehydrogenase gene (*tdh*) deletion mutant. Under *Δtdh* background conditions, we readily obtained an *aao_So_-mutT* double gene mutant; however, no such mutant was ever made from the wild-type strain as mentioned above. Again this demonstrates that threonine-derived aminoacetone is the deleterious compound that needs LAAO to remove. In addition, the triple mutant *Δtdh-Δaao_So_-ΔmutT* exhibited a growth reduction of only 10% relative to the wild-type strain (Fig. S6). This suggests that the partial growth impairment of *Δaao_So_-mutT* strain is caused by the accumulation of aminoacetone.

**Table 3:** Effect of aminoacetone on the growth of *S. oligofermentans* wild-type and two mutants.

Growth mass (OD_600_ )*						
	wild-type		*Δaao_So_*		*Δaao_So_-mutT*	
Growth time	−	+	−	+	−	+
6 h	0.46±0.01	0.51±0.02	0.44±0.02	0.49±0.03	0.33±0.01	0.29±0.02
8 h	0.98±0.03	1.05±0.03	0.96±0.03	0.96±0.04	0.78±0.01	0.71±0.01

* Symbol: +, 5 mM aminoacetone added; -, no aminoacetone added; Data are shown as means±SD from three independent experiments.

## Discussion

In this study, we used genetic and biochemical approaches to demonstrate that the gene operon *aao_So_-mutT* constitutes a dual safeguard mechanism, protecting cells from ROS stress in *S. oligofermentans*. Using *aao_So_*-encoded aminoacetone oxidase, cells can reduce the generation of aminoacetone-derived superoxide anion, and the coexpressed MutT eliminates the mutagens derived from ROS. Concerted action of the two proteins can be modulated by the equivalent expression of two genes under the control of a single promoter. This anti-ROS defense mechanism may also be employed by other streptococci. A similar gene operon has also been found in *Streptococcus pneumoniae*, a streptococcus inhabiting in human upper respiratory tract [25]. However, this operon has not yet been found in other lactic acid bacteria whose genomes are available.

Previous research has shown that aminoacetone, a prooxidant, rapidly reacts with oxygen through a superoxide-propagated mechanism even in the presence of strong chelators [26], while semicarbazide-sensitive amine oxidase (SSAO) functions as a cleaner of aminoacetone in human cells [15]. Considering that (1) both LAAO and SSAO belong to the family of amine oxidases; (2) amino acids can also be classified as amines; and (3) aminoacetone is an analog of amino acids; we tested the aminoacetone oxidase activity of LAAO. Pronounced aminoacetone oxidase activity was detected for LAAO, although it was first identified as an amino acid oxidase [11]. Cellular aminoacetone can be derived either from threonine or a condensation of glycine and acetyl-CoA [24], [27]. Threonine dehydrogenase, a key enzyme in L-threonine metabolism, catalyzes the conversion of L-threonine to 2-amino-3-ketobutyrate, and the latter is spontaneously decarboxylated to aminoacetone [28]. No gene homology of 2-amino-3-ketobutyrate coenzyme A ligase, which catalyzes glycine and acetyl-CoA in the formation of 2-amino-3-ketobutyrate, was found in the genome of *S. oligofermentans*. However, the *tdh* gene was present and an elevated transcription level was detected in both *Δaao_So_-mutT* and *Δaao_So_* mutants (data not shown). This increased aminoacetone production and decreased scavenging. In addition, the *aao_So_-mutT* double gene mutant was only obtained using a background of *tdh* mutation, indicating that aminoacetone is derived from threonine can accumulate to lethal levels when both *aao_So_* and *mutT* are absent. Because aminoacetone can react with transition metals, it does not accumulate to a great extent in the cells. This is also true of human cells [23]. The reason why *aao_So_* inactivation induces expression of *tdh* remains unknown.

It is known that ROS can oxidize nucleotides [19], [29]. Among nucleotides, dGTP is most susceptible to oxidation, especially to hydroxyl radicals, and the oxidized product 8-oxo-dGTP causes mutagenesis and carcinogenesis [18], [19]. Organisms have evolved many different approaches to dealing with mutagenesis. These include the MutT protein in *E. coli* and hMTH-1 protein in humans, both of which are known to hydrolyze 8-oxo-dGTP to the non-harmful 8-oxo-dGMP and PPi [19], [30]. MutT belongs to the Nudix hydrolase superfamily, which requires Mg^2+^. These enzymes hydrolyze the nucleoside diphosphate modified with other moieties. The most suitable substrate for *E. coli* MutT is 8-oxo-dGTP [31]. In the present study, similar types of activity were determined for *S. oligofermentans* MutT. The high levels of expression of *mutT* in living *S. oligofermentans* cells and the enzymatic activity of MutT suggest that *S. oligofermentans* MutT also plays a role in the elimination of deleterious oxidized nucleotides.

**Figure 4:**
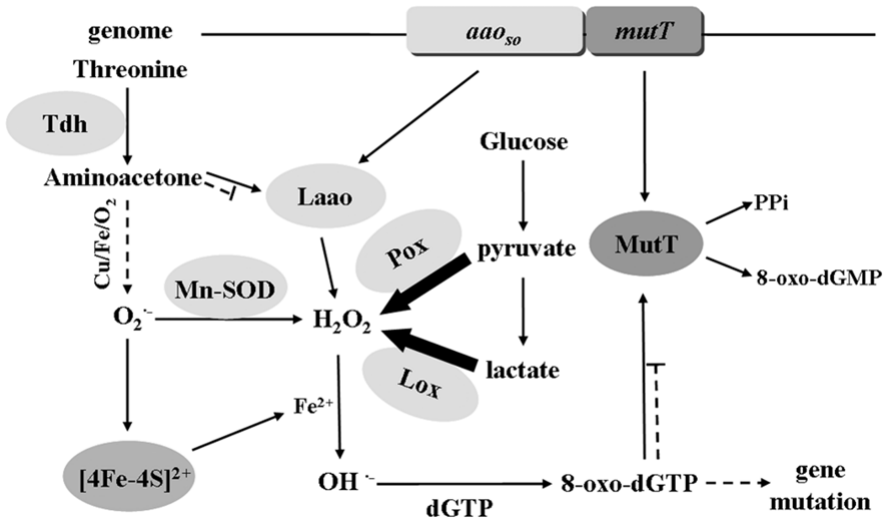
Depicted concerted working model of LAAO and MutT in preventing ROS-generated oxidative damage to cells. *aao_So_* and *mutT* constitute an operon in *S. oligofermentans* and are co-transcribed, creating the equivalent protein products of the two. The absence of LAAO, an aminoacetone oxidase, was found to cause the accumulation of aminoacetone, which can be derived from threonine by threonine dehydrogenase (Tdh). It generates superoxide anions in the presence of oxygen and transition metals. Fe^2+^, released from proteins containing [4Fe–4S]^2+^ cluster by superoxide anion attack, was found to trigger the Fenton reaction to form hydroxyl radicals from H_2_O_2_, which is produced in abundance from lactate and pyruvate by Lox and Pox, respectively. These hydroxyl radicals oxidize nucleic acids, like dGTP to 8-oxo-dGTP, a mutagen that can cause severe gene mutations. The coexpressed MutT hydrolyzes 8-oxo-dGTP to the harmless 8-oxo-dGMP, and so prevents cell damage. LAAO, L-amino acid oxidase; MutT, pyrophosphohydrolase; Tdh, threonine dehydrogenase; Lox, lactate oxidase; Pox, pyruvate oxidase; Mn-SOD, manganese-dependent superoxide dismutase. Thick arrows refer to the main flux of H_2_O_2_; broken arrows indicate the conditional pathways.

Although the *Δaao_So_* mutant generates hydroxyl radicals from superoxide anion and H_2_O_2_, the intact *mutT* may be sufficient to the scavenging nucleotide oxides generated by hydroxyl radicals. This may be why no impairment of growth was observed. Under normal growth conditions, the production of ROS is limited to a very low level, thus, *ΔmutT* did not show any detectable reduction in growth. However, inactivation of both *aao_So_* and *mutT* is lethal for *S. oligofermentans*. Therefore, synergy of LAAO and MutT is essential for bacterial defense against ROS damage. Here, we propose a working model of LAAO and MutT in prevention of ROS-generated cell oxidative damage (Fig. 4).

The *aao_So_*-*mutT* operon and possible coexpression are found in the genome of *S. pneumoniae* (GenBank accession No. NC_008533). This clinical streptococcus resembles *S. oligofermentans* with respect to H_2_O_2_ production and tolerance [10], [25]. Like *S. pneumoniae*, which lives in an oxygen-rich niche in the upper respiratory tract, *S. oligofermentans* inhabits the human oral cavity, where the oxygen content fluctuates [25], [32], [33]. In this way, the *aao_So_-mutT* operon may have been the evolutionarily selected by bacteria living in environments rich in oxygen.

## Materials and Methods

### Bacterial strains and Culture Conditions

*S. oligofermentans* strain AS 1.3089^T^ and the *aao_So_*-insertional mutant were cultured in our laboratory [9], [11]. Strains were routinely cultured in TYG broth at 37°C in static culture [34]. Agar plates were cultured in a candle jar. Brain heart infusion (BHI) medium (Becton Dickinson, U.S.) supplemented with spectinomycin (800 µg ml^−1^) or kanamycin (500 µg ml^−1^) was used for selection of antibiotic-resistant colonies. *E. coli* DH5α (Transgen Biotech, Beijing, China) and BL21 (Novagen, Germany) were grown in Luria–Bertani (LB) broth or on LB agar plate aerobically at 37°C. They were used for plasmid amplification and protein overexpression. Transformants were selected in LB medium with ampicillin (100 µg ml^–1^), kanamycin (50 µg ml^–1^), or spectinomycin (250 µg ml^−1^).

### Construction of *aao_So_*, *mutT*, and *sodA* Gene Deletion Mutants

The genome DNA of *S. oligofermentans* was extracted and purified as described previously [33], [35]. PCR primers listed in Table 4 were designed according to the genome draft of *S. oligofermentans* and synthesized by Sangon Company (Shanghai, China). The deletion mutants of the *aao_So_*, *mutT*, and *sodA* genes were constructed using a PCR ligation method [36]. Two∼600 bp sequences upstream and downstream of each of the three genes were PCR amplified using the primers listed in Table 4. PCR products were digested with *Bam*HI (New England Biolabs, U.S.) and a non-polar kanamycin resistance gene cassette was cut from plasmid pALH124 with *Bam*HI as well [36]. The three fragments at equivalent molar ratios were ligated using T_4_ DNA ligase (Fermentas, Canada). The fused fragment was transformed into wild-type *S. oligofermentans* with the assistance of a chemical synthetic *S. oligofermentans*- specific competence-stimulating peptide (CSP) [33]. Briefly, overnight culture of *S. oligofermentans* was diluted 1∶30 into fresh BHI broth (with 0.4% BSA) and cultured at 37°C for 30 min. The fused fragment was added at a final concentration of 500 µg ml^−1^ together with the CSP (final concentration 160 ng ml^−1^). After 5 h incubation, the mixture was plated on BHI agar containing the selective antibiotics and incubated at 37°C in a candle jar. A *sodA* deletion of *Δaao_So_-mutT* mutant was constructed using the same method. Positive transformants of the *Δaao_So_-mutT-ΔsodA* mutant were selected on BHI agar plates containing 500 µg ml^–1^ kanamycin and 800 µg ml^−1^ spectinomycin. Transformants of *Δaao_So_*, *ΔmutT*, and *ΔsodA* were selected with 500 µg ml^−1^ each of kanamycin. They were all confirmed by PCR.

**Table 4:** Primers used in this study.

Primer	Sequence*	Target genes
spacer-lm-F	TCTCCACTTTGCCGGTGA	spacer between *aao_So_* and *mutT*
spacer-lm-R	CATGCACATGTTGACAAATTC	
spacer-neg-F	TCATCAAATAATTGGGCAAGC	negative control for space check
spacer-neg-R	ACAAACTATTGACAGCGCTATCAC	
qPCR-16S-F	GGCGTGCCTAATACA	qPCR for 16S rRNA
qPCR-16S-R	AGACTTCCGTCCATT	
qPCR-Sod-F	ATGGCAATTATTTTACCAGATCTAC	qPCR for *sodA*
qPCR-Sod-R	TTCTTCGAATGAGCCAAATG	
qPCR-mutT-F	GAATTTGTCAACATGTGCATGA	qPCR for *mutT*
qPCR-mutT-R	CGGTTGCAAGTTTCAGATGA	
qPCR-mutX-F	ATTCGTAAAAATCAACCTGCG	qPCR for *mutX*
qPCR-mutX-R	TTTCCTGAGTTTACTCCCGA	
mutT-kana-up-F	GAAGAAAAAGGAGAAAGAAAG	upstream fragment of *mutT* for deletion
mutT-kana-up-R	AAGGATCCAAAATGAGAGTGGGACAG	
mutT-kana-down-F	AAGGATCCCTTGTTCTCCCTTTAAAT	downstream fragment of *mutT* for deletion
mutT-kana-down-R	TGCTGAAAGTCCTCTTTTGAC	
*aao_So_* -kana-up-F	ATGAACCATTTCGACACAATC	upstream fragment of *aao_So_* for deletion
*aao_So_* -kana-up-R	TTGGATCCTGTCATGGTTGTCAAAC	
*aao_So_* -kana-down-F	TTGGATCCAGGCCCTCATCAAGCAAA	downstream fragment of *aao_So_* for deletion
*aao_So_* -kana-down-R	TTAATCATAATGCAAACTTC	
pDL-lm-F	ATGGATCCTAAAAAATGAGCCTAAAATC	construction of shuttle plasmid
pDL-lm-R	TATGTCGACTTTTTACTTCAAGTCATAAA	
lm-kana-up-F	GAAGAAAAAGGAGAAAGAAAG	upstream fragment of *aao_So_-mutT* for deletion
lm-kana-up-R	AAGGATCCAAAATGAGAGTGGGACAG	
lm-kana-down-F	AAGGATCCGTATCTTTTCCTTTCGTG	downstream fragment of *aao_So_-mutT* for deletion
lm-kana-down-R	CAGAGGTCAAGCAGCCCTTG	
tdh-add9-up-F	GGATGCGACTGGCAATGCCAAAGAT	upstream fragment of *tdh* for deletion
tdh-add9-up-R	TTAAGCTTTCCTTTTCCTTTCCAAATGAG	
tdh-add9-down-F	CTTGACTTCTGCATCTGGCTCAAC	downstream fragment of *tdh* for deletion
tdh-add9-down-R	TTTCCCGGGTCCTAAAAATATTTTAGC	
sod-kana-up-F	GGTTTGCCCAGTCGTCAGGTC	upstream fragment of *sodA* for deletion
sod-kana-up-R	TTGGATCCGAACTCTACGCAGC	
sod-kana-down-F	TTGGATCCGATCTGGTAAAATAA	downstream fragment of *sodA* for deletion
sod-kana-down-R	CAATATTTTTGATTTGACCCAGCTG	
pET-mutT-F	TTCCATGGGGATGAACAGACGCGAAACC	overexpression of MutT
pET-mutT-R	TTCTCGAGCTTCAAGTCATAAAGC	

* Underlined sequences indicate the restriction enzyme sites. All primers are original in this study.

### Construction of *aao_So_-mutT* and *tdh-aao_So_-mutT* Deletion Mutants

To delete the *aao_So_-mutT* genes on the chromosome, we first constructed a shuttle plasmid, pDL278-*aao_So_-mutT*. The entire *aao_So_-mutT* operon fragment (including the promoter region) was amplified with paired primers pDL-lm-F and pDL-lm-R (Table 4). The fragment was then digested with *Bam*HI and *Sal*I and inserted into plasmid pDL278, which was digested with the same enzymes. The recombinant plasmid was transformed into the wild-type strain of *S. oligofermentans*, and the positive strain was selected on BHI agar containing spectinomycin (800 µg ml^−1^). Upon PCR confirmation of the introduced plasmid-carried *aao_So_*-*mutT* operon, a deletion of *aao_So_*-*mutT* operon on the chromosome was conducted using the same method as the single gene mutation above except that the positive transformants were selected on BHI agar plates containing 500 µg ml^−1^ kanamycin and 800 µg ml^−1^ spectinomycin.

To produce the triple-gene *tdh-aao_So_-mutT* deletion mutant, the *tdh* gene was knocked out to stop the generation of aminoacetone. Briefly, two∼600 bp sequences upstream and downstream of the *tdh* gene were PCR amplified using the corresponding primers listed in Table 4. PCR products were double digested with *Hind*III and *Sma*I (New England Biolabs, U.S.) and a spectinomycin resistance gene cassette was cut from plasmid pFW5-luc with *Hind*III and *Sma*I [33], [37]. The three fragments at equivalent molar ratio were ligated using T_4_ DNA ligase (Fermentas, Canada), and the fused fragment was transformed into wild-type *S. oligofermentans*. *Δtdh* mutant was selected on BHI agar plate containing 800 µg ml^−1^ spectinomycin. Under *Δtdh* background, the *aao_So_-mutT* operon was knocked out. The triple gene deletion *Δtdh-Δaao_So_-ΔmutT* was selected on BHI agar plate containing 500 µg ml^−1^ kanamycin and 800 µg ml^–1^ spectinomycin. The positive clones were confirmed by PCR.

### Construction of *aao_So_-mutT* Gene Complementary Strain

The *aao_So_-mutT* coding region and its inherent promoter were amplified using the paired primers pDL-lm-F and pDL-lm-R (Table 4). The PCR product was double digested with *Bam*HI and *Sal*I and inserted into plasmid pDL278, which was digested with the same enzymes. Recombinant plasmid pDL278-*aao_So_-mutT* was transformed into the *Δaao_So_-mutT* mutant; transformants were selected on BHI agar containing spectinomycin (5 mg ml^−1^) under aerobic conditions. Positive transformants were confirmed by PCR and sequencing.

### Quantification of Gene Expression with Quantitative PCR

Total RNA was extracted using TRIzol reagent (Invitrogen, U.S.) according to the supplier’s recommendations. Cells were harvested at the middle logarithmic phase, washed with RNase-free water, and frozen in liquid nitrogen, and then ground three times with a glass rod. Then TRIzol reagent was added. After incubation at room temperature for 5 min, chloroform was mixed with the lysate and the mixture centrifuged at 12,000 ×g. RNA in the aqueous phase was then precipitated using isopropyl alcohol, washed with 75% ethanol, and air dried for 15 min. The pellet was then dissolved in 30 µl RNase-free water, and trace amounts of DNA were removed by DNase. The concentration and quality of RNA samples were determined using NanoDrop spectrophotometer and gel electrophoresis. cDNA was synthesized from 2 µg RNA according to manufacturer’s instructions (Promega, U.S.) and used for quantitative PCR amplification with the corresponding primers listed in Table 4. Amplifications were performed with ABI PRISM 7000 Sequence Detection System (Applied Biosystems, U.S.). The thermocycling parameters included one cycle of 95°C for 10 s, followed by 40 cycles of 95°C for 5 s, 51°C for 30 s, and 72°C for 30 s. Transcripts were quantified with the comparative threshold cycle (Ct) values. To estimate the copy numbers for a given mRNA, a standard curve of the tested gene was generated by cloning the corresponding PCR fragment at a length of about 260 bp into pMD-18T vector (TaKaRa, Japan). The plasmid was then serially diluted and used to generate the standard curve of quantitative PCR. The 16S rRNA gene was used as the biomass reference. The copy number of each gene was normalized to 16S rRNA copies. The copies of the transcript of each gene are shown as per 10,000 16S rRNA copies.

### Overexpression and Purification of MutT Protein

The *mutT* gene of *S. oligofermentans* was amplified using a pair of primers (Table 4). Pyrobest DNA polymerase (TaKaRa, Japan) was used for PCR amplification of *mutT*. A 453 bp fragment was subsequently digested with *Nco*I-*Xho*I (New England Biolabs, U.S.) and cloned into the *Nco*I-*Xho*I restriction sites of the expression vector pET-28a (Novagen, Germany). The His6-tag fusion sites and nucleotide sequences of the double-stranded template DNA were confirmed by sequencing.

The pET-28a plasmid carrying the *mutT* gene was transformed into *E. coli* BL21 (DE3) pLysS (Novagen, Germany) and cultured in LB medium supplemented with 50 µg ml^−1^ kanamycin. Cells were grown at 37°C to OD_600_ 0.4–0.6. Overexpression of MutT protein was induced by addition of 1 mM isopropyl-β-D- thiogalactopyranoside. The culture was incubated for an additional 3 h before harvesting. Cells were collected by centrifugation at 10,000×g for 10 min at 4°C, resuspended in the binding buffer (20 mM sodium phosphate, 0.5 M NaCl, 40 mM imidazole, pH 7.4), and was sonicated for 20 min. After the cell lysate was spun down at 10,000 ×g for 10 min at 4°C, the supernatant was filtered through a 0.22 µm polyvinylidene difluoride membrane (Millipore, U.S.) and then applied to a Histrap column (GE Healthcare, USA) previously equilibrated with the binding buffer. Proteins were eluted by elution buffer (20 mM sodium phosphate, 0.5 M NaCl, 500 mM imidazole, pH 7.4), and fractions of the elution were subjected to 12% SDS-PAGE. The desired protein fractions were desalted by loading onto HiPrep™ 26/10 Desalting (GE Healthcare, U.S.) column and eluted with 20 mM Tris–HCl buffer (pH 9.0) with 10% glycerol. The purified MutT protein was stored at –80°C.

### Assay of Aminoacetone Oxidase Activity for LAAO

The aminoacetone activity of LAAO was determined by measuring H_2_O_2_ production via horseradish peroxidase (HRP)-coupled assay [11]. The assay mixture (1 ml) contained 200 nM LAAO and 25 mM aminoacetone (Toronto Research Chemicals, Canada) in 20 mM Tris–HCl buffer (pH 8.0). Lysine at concentrations of 25 mM and 100 mM was used as the control. The reaction mixture was incubated at 37°C for 30 min. H_2_O_2_ was quantified using a modified version of a previously described method [11], [38]. Briefly, 650 µl of reaction supernatant was added to 600 µl of the solution containing 2.5 mM 4-amino-antipyrine (4-amino-2, 3-dimethyl-1-phenyl- 3-pyrazolin-5-one; Sigma–Aldrich, U.S.) and 0.17 M phenol. The reaction proceeded for 4 min at room temperature, and HRP (Sigma–Aldrich, U.S.) was added at a final concentration of 50 mU ml^−1^ in 200 mM potassium phosphate buffer (pH 7.2). After 4 min of incubation at room temperature, OD_510_ was measured using a Unico 2100 visible spectrophotometer (Shanghai, China). A standard curve was generated with H_2_O_2_.

### Mn-SOD Activity Assay

The Mn-SOD activity of *S. oligofermentans* was determined using the method of inhibition of pyrogallol auto-oxidation [39], [40]. The reaction mixtures included 4.5 ml 50 mM Tris–HCl (pH 8.2), 1 mM EDTA, 0.1 mM pyrogallol, and 10 µl freshly prepared lysate supernatant of the strains. The same volume of 10 mM HCl was used as a blank control. After mixing, OD_325_ was read every 30 s for 4 min at 25°C using a Unico 2100 visible spectrophotometer. The OD_325_ increase rate was calculated from 0 to 4 min. One unit of enzymatic activity was defined as the amount of enzyme used for 50% reduction of pyrogallol auto-oxidation rate per min per mg protein.

### *S. oligofermentans* MutT Activity Assay

The enzymatic activity of *S. oligofermentans* MutT was determined based on the 8-oxo-dGTP degradation. The assay mixture contained 100 µl 100 mM Tris–HCl (pH 9.0), 5 mM MgCl_2_, 100 µM 8-oxo-dGTP (eENZYME, U.S.), 5% glycerol and 100 nM MutT protein. Desalting buffer without enzyme was used as a negative control. The reaction mixtures were incubated at 37°C for 20 min, and terminated by adding 50 mM EDTA (EDTA: Mg^2+^  = 3.3∶ 1). Twenty microliters of sample was analyzed on HPLC with the following parameters, flow phases: 20 mM sodium phosphate buffer (pH 6.0) and acetonitrile (95∶5); column temperature 30°C; flow rate 0.6 ml min^–1^. The UV absorbance of nucleotide was detected at 293 nm.

### Determination of Cellular Aminoacetone Derivate

Cells of *S. oligofermentans* wild-type and two mutants at their mid-log phase were collected and washed twice with 100 mM sodium phosphate buffer (pH 7.3) by centrifugation at 12,000 ×g for 10 min. Fifty milligrams of pelleted cells of each culture were resuspended in 1 ml 100 mM phosphate buffer (pH 7.3), then homogenized in a MINI Bead Beater (Biospec Products; Bartlesville, OK, U.S.) for 20 s, with a 2-min interval on ice. This process was repeated five times. After centrifugation of the cell extract at 12,000×g for 10 min at 4°C, the supernatant was partially purified using a 3 KD ultrafiltration tube (MWCO, Millipore, U.S.). Synthetic aminoacetone (Toronto Research Chemicals, Canada) and the cell extract samples were treated with FMOC-Cl to obtain the aminoacetone derivate using a modified version of the method described by Kazachkov and Yu [23]. Briefly, 1 ml sodium borate buffer (0.4 M, pH 10) was added to 1 ml of samples or aminoacetone (10 mM) and vortexed for 1 min. Then, 1 ml FMOC-Cl reagent (10 mM in acetonitrile) was mixed with the samples by vortex for 1 min. Five milliliters of hexane was added to terminate the reaction by extracting the excess reagent (FMOC-Cl), hydrolysis product FMOC-OH and acetonitrile. The upper hexane layer was discarded and the process was repeated twice, and the remaining solution was neutralized by addition of 100 µl 20% (v/v) acetic acid for HPLC assay using a modified version of the method described by Xiao and Yu [41]. The assay was performed on Shimadzu HPLC system (Japan) with a C_18_ reverse phase column (4.6×250 mm/5 µm; Agilent, U.S.), and the compound separation was achieved under the following conditions, flow phases: 5 µM potassium phosphate buffer (pH 4.40–4.45) and acetonitrile (60∶40), column temperature 30°C; flow rate 1 ml min^−1^. FMOC–aminoacetone was detected for UV absorbance at 265 nm.

### Determination of ROS Levels

Cellular ROS levels in *S. oligofermentans* were measured using a spin trapping agent combined with ESR spectroscopy (Bruker ESP 300, Germany) [21], [22]. 5,5-Dimethyl-l-pyrroline *N*-oxide (DMPO, TCI, Japan) was purified by stirring a solution (0.25 M) of the spin trap in deionized water with a little activated charcoal for 1 hour. After filtration, this solution was stored in the dark at -20°C. The wild-type strain and two mutants were collected at mid-log phase and washed twice with sodium sulfate solution (50 mM). The standard incubation consisted of the following final concentrations: 30 mg bacterial cells, DMPO solution (100 mM), and each sample were brought up to 0.5 ml with deionized water. The mixtures were incubated at 37°C for 5 min before detection. ESR samples in a quartz flat cell were fixed in the cavity of the ESR spectrometer. ESR measurements were taken at 9.75 GHz with a 100 kHz modulation frequency, and the following ESR spin trapping parameters were used: field sweep, 343.2–353.2 mT; microwave frequency, 9.75 GHz; sweep time, 168 s; microwave power, 12.8 mW; modulation amplitude 1 mT; and time constant, 328 ms.

## Supporting Information

Figure S1 **Growth of the wild-type,** ***Δaao_So_*** **and** ***ΔmutT*** **mutants of** ***S. oligofermentans*** **in glucose.** Strains were pre-cultured overnight in TYG medium, and 1∶100 diluted with fresh TYG medium with the same OD_600_. Samples were taken every 1 h to detect OD_600_. Symbols: △, wild-type strain; ○, *Δaao_So_* mutant; ×, *ΔmutT* mutant. The results are shown as the means±SD of three independent experiments.
(TIF)

Figure S2 **Enzymatic assays for the overexpressed LAAO of** ***S. oligofermentans*** **.** Activities are expressed by H_2_O_2_ production rate as described in materials and methods**.** Data are the means of three assays, and standard deviations are shown.
(TIF)

Figure S3 **Pyrophosphohydrolase activity of** ***S. oligofermentans*** **MutT for 8-oxo-dGTP determined through HPLC.** (A) 8-oxo-dGTP (100 µM) and (B) 8-oxo-dGTP incubated with 100 nM purified MutT at 37°C for 20 min. Retention times for 8-oxo-dGTP and 8-oxo-dGMP are shown.
(TIF)

Figure S4 **Growth of the wild-type,** ***Δaao_So_-mutT*** **,** ***Δsod*** **, and** ***Δaao_So_-mutT-Δsod*** **mutants of** ***S. oligofermentans*** **.** Strains were cultured overnight and adjusted to the same OD_600_, and then 1∶100 diluted with fresh TYG medium and cultured statically unless indicated otherwise. OD_600_ was measured in 1 h interval. Symbols: △, wild-type strain; □, *Δaao_So_-mutT* mutant; ◊, *Δsod* mutant; ×, *Δaao_So_-mutT-Δsod* mutant; ○, *Δaao_So_-mutT-Δsod* mutant growing strict anaerobically. Results represent the means±SD from three independent experiments.
(TIF)

Figure S5 **HPLC chromatogram of FMOC-aminoacetone obtained from the wild-type and both mutants cells of** ***S. oligofermentans*** **.** The FMOC derivate signal of the chemical aminoacetone appears at 27.5 min under the analytical conditions. (A) FMOC derivate of chemical aminoacetone; (B) wild-type strain; (C) *Δaao_So_* mutant, and (D) *Δaao_So_-mutT* mutant.
(TIF)

Figure S6 **Growth of** ***Δtdh-Δaao_So_-ΔmutT*** **mutant and the wild-type of** ***S. oligofermentans*** **.** Strains were cultured overnight and adjusted to the same OD_600_ in TYG medium, and 1∶100 diluted with the same medium. OD_600_ was detected in 1 h interval. Symbols: △, wild-type strain;□, *Δtdh-Δaao_So_-ΔmutT* mutant. The results are shown as the means ± SD of three independent experiments.
(TIF)

## References

[1] Farr SB, Kogoma T (1991). Oxidative stress responses in *Escherichia coli* and *Salmonella typhimurium*.. Microbiol Rev.

[2] Soonsanga S, Lee JW, Helmann JD (2008). Oxidant-dependent switching between reversible and sacrificial oxidation pathways for *Bacillus subtilis* OhrR.. Mol Microbiol.

[3] Spolarics Z, Wu JX (1997). Role of glutathione and catalase in H_2_O_2_ detoxification in LPS-activated hepatic endothelial and Kupffer cells.. Am J Physiol.

[4] Yesilkaya H, Kadioglu A, Gingles N, Alexander JE, Mitchell TJ (2000). Role of manganese-containing superoxide dismutase in oxidative stress and virulence of *Streptococcus pneumoniae*.. Infect Immun.

[5] Jenney FE, Verhagen MF, Cui X, Adams MW (1999). Anaerobic microbes: oxygen detoxification without superoxide dismutase.. Science.

[6] Barre O, Mourlane F, Solioz M (2007). Copper induction of lactate oxidase of *Lactococcus lactis*: a novel metal stress response.. J Bacteriol.

[7] Magnani D, Barre O, Gerber SD, Solioz M (2008). Characterization of the CopR regulon of *Lactococcus lactis* IL1403.. J Bacteriol.

[8] Rodriguez R, Redman R (2005). Balancing the generation and elimination of reactive oxygen species.. Proc Natl Acad Sci U S A.

[9] Tong H, Gao X, Dong X (2003). *Streptococcus oligofermentans* sp. nov., a novel oral isolate from caries-free humans.. Int J Syst Evol Microbiol.

[10] Tong H, Chen W, Merritt J, Qi F, Shi W (2007). *Streptococcus oligofermentans* inhibits *Streptococcus mutans* through conversion of lactic acid into inhibitory H_2_O_2_: a possible counteroffensive strategy for interspecies competition.. Mol Microbiol.

[11] Tong H, Chen W, Shi W, Qi F, Dong X (2008). SO-AAO, a novel L-amino acid oxidase that enables *Streptococcus oligofermentans* to outcompete *Streptococcus mutans* by generating H_2_O_2_ from peptone.. J Bacteriol.

[12] Liu L, Tong H, Dong X (2012). Function of pyruvate oxidase – lactate oxidase cascade in interspecies competition between *Streptococcus oligofermentans* and *Streptococcus mutans*..

[13] Zhu B, Tong H, Chen W, Dong X (2009). Role of *dpr* in hydrogen peroxide tolerance of *Streptococcus oligofermentans*.. Wei Sheng Wu Xue Bao.

[14] Bialkowski K, Kasprzak KS (1998). A novel assay of 8-oxo-2'-deoxyguanosine 5'-triphosphate pyrophosphohydrolase (8-oxo-dGTPase) activity in cultured cells and its use for evaluation of cadmium(II) inhibition of this activity.. Nucleic Acids Res.

[15] Sartori A, Garay-Malpartida HM, Forni MF, Schumacher RI, Dutra F (2008). Aminoacetone, a putative endogenous source of methylglyoxal, causes oxidative stress and death to insulin-producing RINm5f cells.. Chem Res Toxicol.

[16] Deng Y, Yu PH (1999). Assessment of the deamination of aminoacetone, an endogenous substrate for semicarbazide-sensitive amine oxidase.. Anal Biochem.

[17] Buffoni F, Ignesti G (2000). The copper-containing amine oxidases: biochemical aspects and functional role.. Mol Genet Metab.

[18] Kalam MA, Haraguchi K, Chandani S, Loechler EL, Moriya M (2006). Genetic effects of oxidative DNA damages: comparative mutagenesis of the imidazole ring-opened formamidopyrimidines (Fapy lesions) and 8-oxo-purines in simian kidney cells.. Nucleic Acids Res.

[19] Nakabeppu Y, Oka S, Sheng Z, Tsuchimoto D, Sakumi K (2010). Programmed cell death triggered by nucleotide pool damage and its prevention by MutT homolog-1 (MTH1) with oxidized purine nucleoside triphosphatase.. Mutat Res.

[20] Imlay JA (2008). Cellular defenses against superoxide and hydrogen peroxide.. Annu Rev Biochem.

[21] Clapp PA, Davies MJ, French MS, Gilbert BC (1994). The bactericidal action of peroxides; an E.P.R. spin-trapping study.. Free Radic Res.

[22] Zhou N, Qiu T, Liu YP, Liu Y (2006). Superoxide anion radical generation in the NaOH/H_2_O_2_/Fe(III) system: a spin trapping ESR study.. Magn Reson Chem.

[23] Kazachkov M, Yu PH (2005). A novel HPLC procedure for detection and quantification of aminoacetone, a precursor of methylglyoxal, in biological samples.. J Chromatogr B Analyt Technol Biomed Life Sci.

[24] Hiraku Y, Sugimoto J, Yamaguchi T, Kawanishi S (1999). Oxidative DNA damage induced by aminoacetone, an amino acid metabolite.. Arch Biochem Biophys.

[25] Pericone CD, Bae D, Shchepetov M, McCool T, Weiser JN (2002). Short-sequence tandem and nontandem DNA repeats and endogenous hydrogen peroxide production contribute to genetic instability of *Streptococcus pneumoniae*.. J Bacteriol.

[26] Dutra F, Knudsen FS, Curi D, Bechara EJ (2001). Aerobic oxidation of aminoacetone, a threonine catabolite: iron catalysis and coupled iron release from ferritin.. Chem Res Toxicol.

[27] Marcus JP, Dekker EE (1993). Identity and some properties of the L-threonine aldolase activity manifested by pure 2-amino-3-ketobutyrate ligase of *Escherichia coli*.. Biochim Biophys Acta.

[28] Bashir Q, Rashid N, Jamil F, Imanaka T, Akhtar M (2009). Highly thermostable L-threonine dehydrogenase from the hyperthermophilic archaeon *Thermococcus kodakaraensis*.. J Biochem.

[29] Taddei F, Hayakawa H, Bouton M, Cirinesi A, Matic I (1997). Counteraction by MutT protein of transcriptional errors caused by oxidative damage.. Science.

[30] Tajiri T, Maki H, Sekiguchi M (1995). Functional cooperation of MutT, MutM and MutY proteins in preventing mutations caused by spontaneous oxidation of guanine nucleotide in *Escherichia coli*.. Mutat Res.

[31] Mildvan AS, Xia Z, Azurmendi HF, Saraswat V, Legler PM (2005). Structures and mechanisms of Nudix hydrolases.. Arch Biochem Biophys.

[32] Marriott HM, Dockrell DH (2006). Streptococcus pneumoniae: the role of apoptosis in host defense and pathogenesis.. Int J Biochem Cell Biol.

[33] Tong H, Zhu B, Chen W, Qi F, Shi W (2006). Establishing a genetic system for ecological studies of *Streptococcus oligofermentans*.. FEMS Microbiol Lett.

[34] Scardovi V (1986). Bergey’s Manual of Systematic Bacteriology..

[35] Dong X, Xin Y, Jian W, Liu X, Ling D (2000). *Bifidobacterium thermacidophilum* sp. nov., isolated from an anaerobic digester.. Int J Syst Evol Microbiol 50 Pt.

[36] Ahn SJ, Wen ZT, Burne RA (2006). Multilevel control of competence development and stress tolerance in *Streptococcus mutans* UA159.. Infect Immun.

[37] Podbielski A, Woischnik M, Leonard BA, Schmidt KH (1999). Characterization of nra, a global negative regulator gene in group A streptococci.. Mol Microbiol.

[38] Seki M, Iida K, Saito M, Nakayama H, Yoshida S (2004). Hydrogen peroxide production in *Streptococcus pyogenes*: involvement of lactate oxidase and coupling with aerobic utilization of lactate.. J Bacteriol.

[39] Scorei R, Cimpoiasu VM, Iordachescu D (2005). In vitro evaluation of the antioxidant activity of calcium fructoborate.. Biol Trace Elem Res.

[40] Siegel D, Gustafson DL, Dehn DL, Han JY, Boonchoong P (2004). NAD(P)H:quinone oxidoreductase 1: role as a superoxide scavenger.. Mol Pharmacol.

[41] Xiao S, Yu PH (2009). A fluorometric high-performance liquid chromatography procedure for simultaneous determination of methylamine and aminoacetone in blood and tissues.. Anal Biochem.

