# Supplementary figures and images for: Role of Operon aaoSo-mutT in Antioxidant Defense in Streptococcus oligofermentans
Source: PLoS One. 2012 May 30;7(5):e38133. doi: 10.1371/journal.pone.0038133 (PMC3364214; doi:10.1371/journal.pone.0038133)

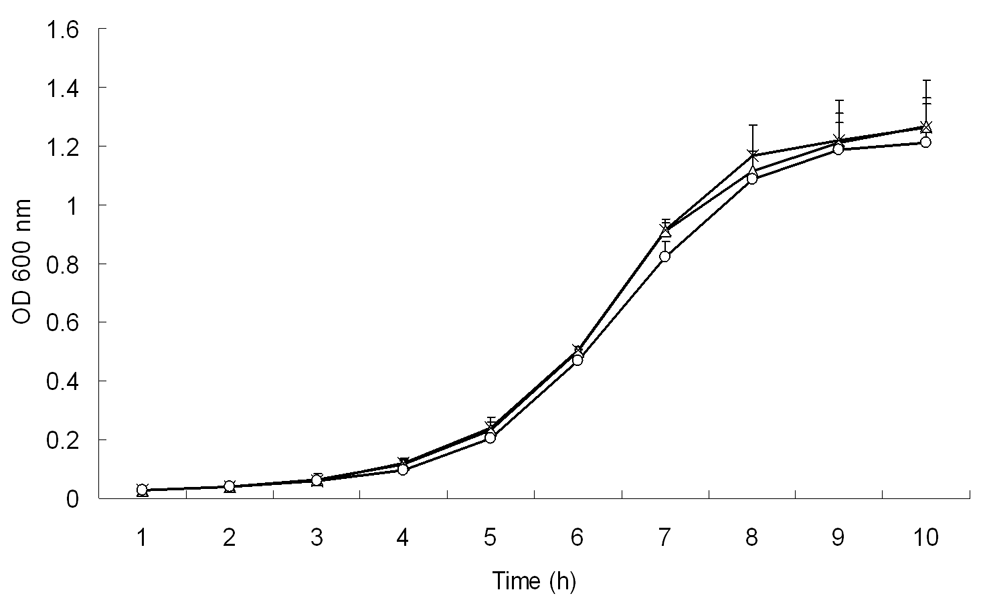

Supplement: Figure S1 — Growth of the wild-type, ΔaaoSo and ΔmutT mutants of S. oligofermentans in glucose. Strains were pre-cultured overnight in TYG medium, and 1∶100 diluted with fresh TYG medium with the same OD600. Samples were taken every 1 h to detect OD600. Symbols: △, wild-type strain; ○, ΔaaoSo mutant; ×, ΔmutT mutant. The results are shown as the means±SD of three independent experiments. (TIF) [file pone.0038133.s001.tif]

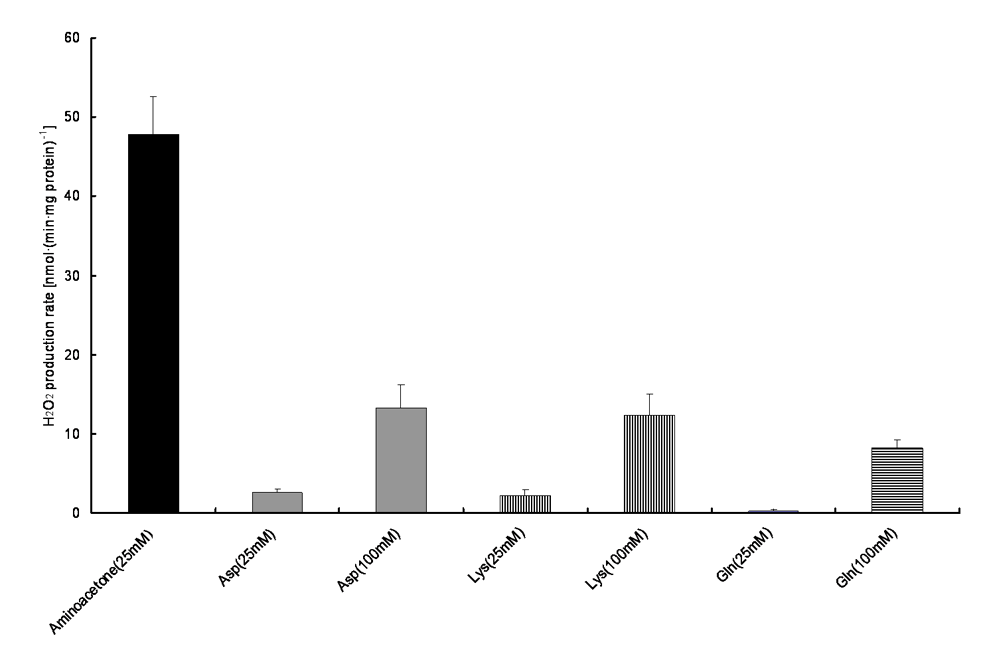

Supplement: Figure S2 — Enzymatic assays for the overexpressed LAAO of S. oligofermentans . Activities are expressed by H2O2 production rate as described in materials and methods. Data are the means of three assays, and standard deviations are shown. (TIF) [file pone.0038133.s002.tif]

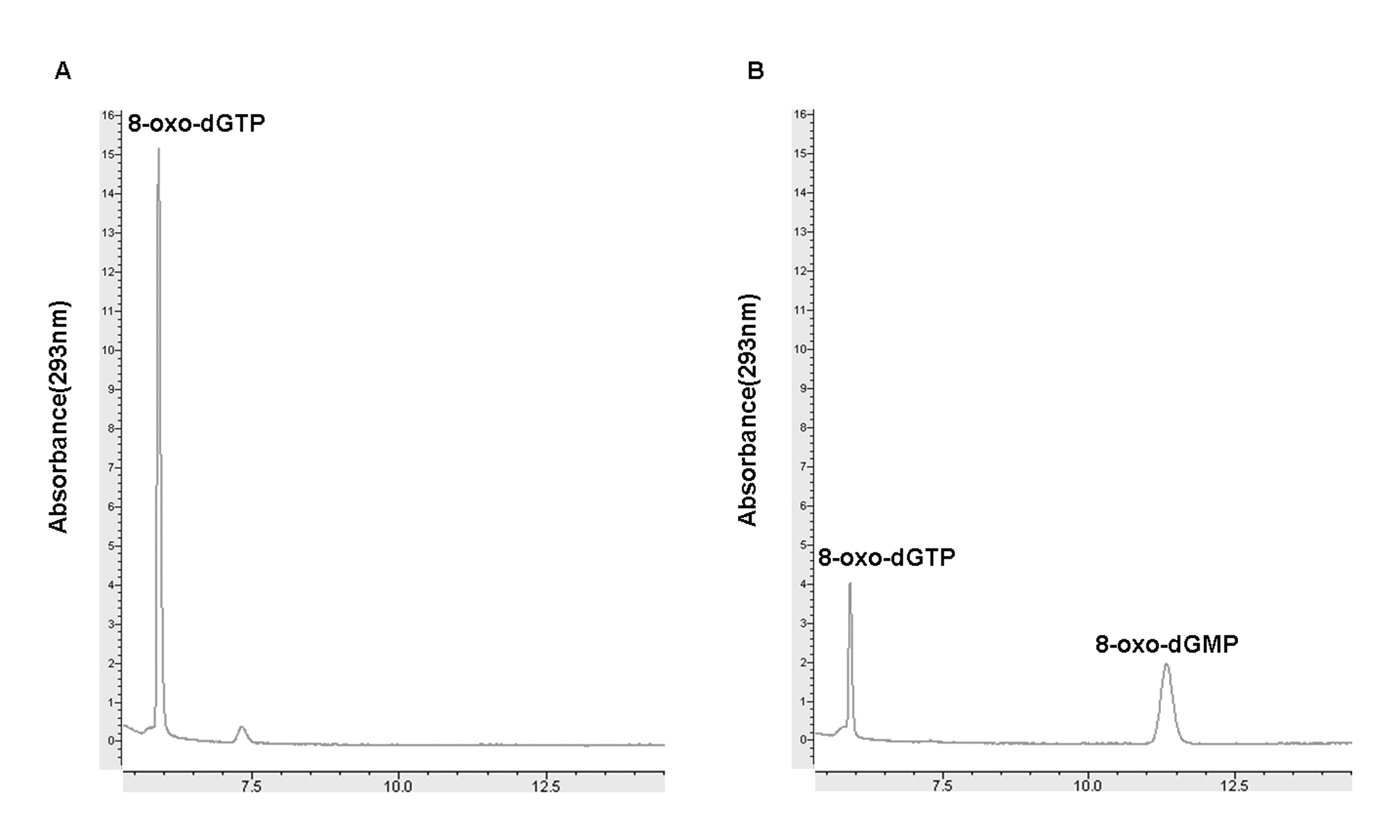

Supplement: Figure S3 — Pyrophosphohydrolase activity of S. oligofermentans MutT for 8-oxo-dGTP determined through HPLC. (A) 8-oxo-dGTP (100 µM) and (B) 8-oxo-dGTP incubated with 100 nM purified MutT at 37°C for 20 min. Retention times for 8-oxo-dGTP and 8-oxo-dGMP are shown. (TIF) [file pone.0038133.s003.tif]

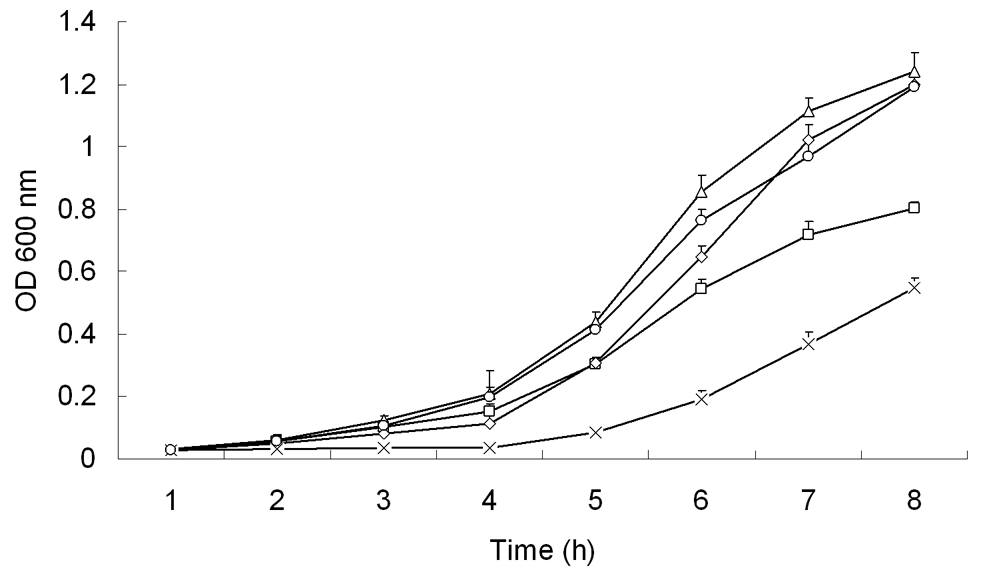

Supplement: Figure S4 — Growth of the wild-type, ΔaaoSo-mutT , Δsod , and ΔaaoSo-mutT-Δsod mutants of S. oligofermentans . Strains were cultured overnight and adjusted to the same OD600, and then 1∶100 diluted with fresh TYG medium and cultured statically unless indicated otherwise. OD600 was measured in 1 h interval. Symbols: △, wild-type strain; □, ΔaaoSo-mutT mutant; ◊, Δsod mutant; ×, ΔaaoSo-mutT-Δsod mutant; ○, ΔaaoSo-mutT-Δsod mutant growing strict anaerobically. Results represent the means±SD from three independent experiments. (TIF) [file pone.0038133.s004.tif]

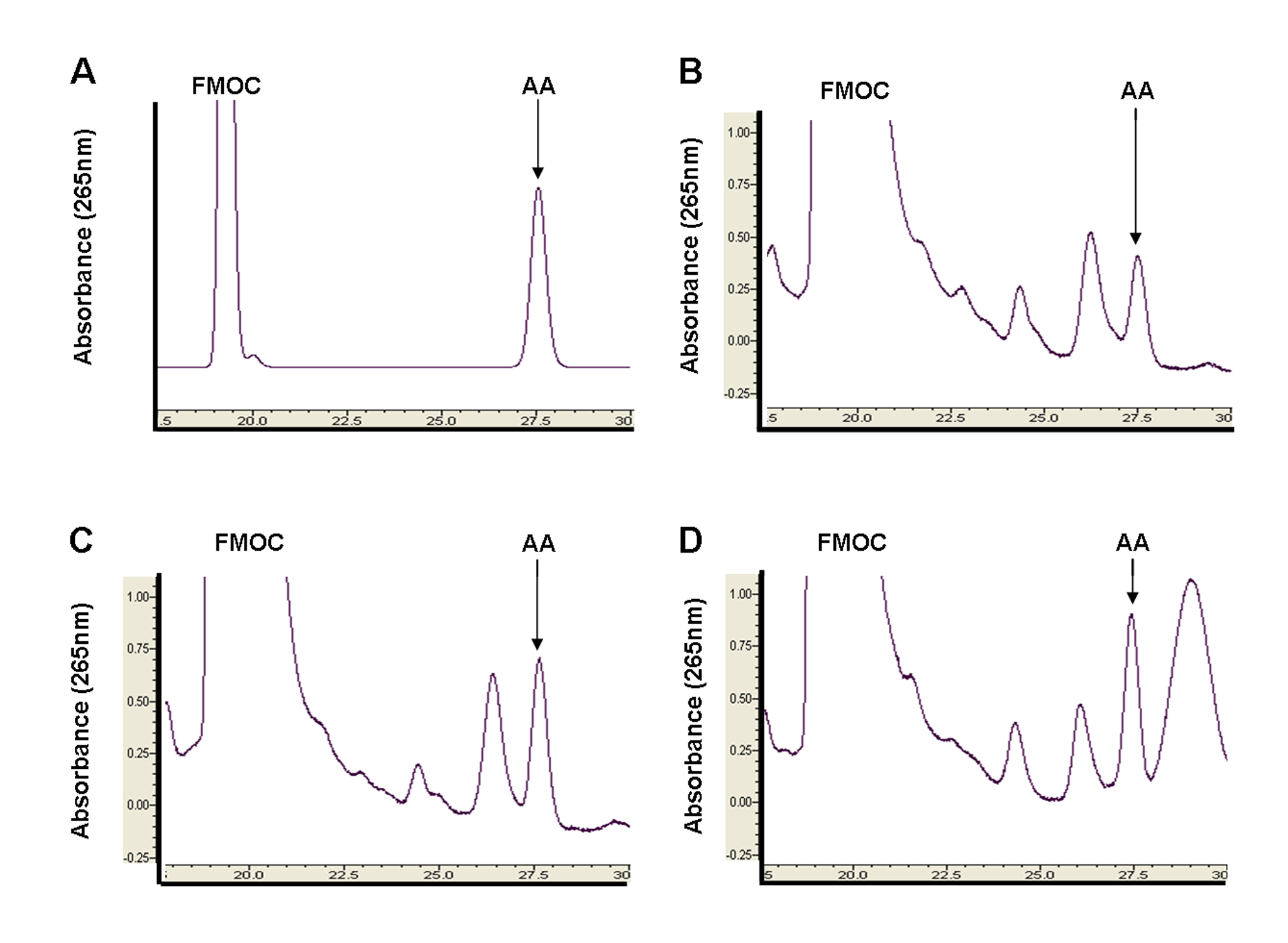

Supplement: Figure S5 — HPLC chromatogram of FMOC-aminoacetone obtained from the wild-type and both mutants cells of S. oligofermentans . The FMOC derivate signal of the chemical aminoacetone appears at 27.5 min under the analytical conditions. (A) FMOC derivate of chemical aminoacetone; (B) wild-type strain; (C) ΔaaoSo mutant, and (D) ΔaaoSo-mutT mutant. (TIF) [file pone.0038133.s005.tif]

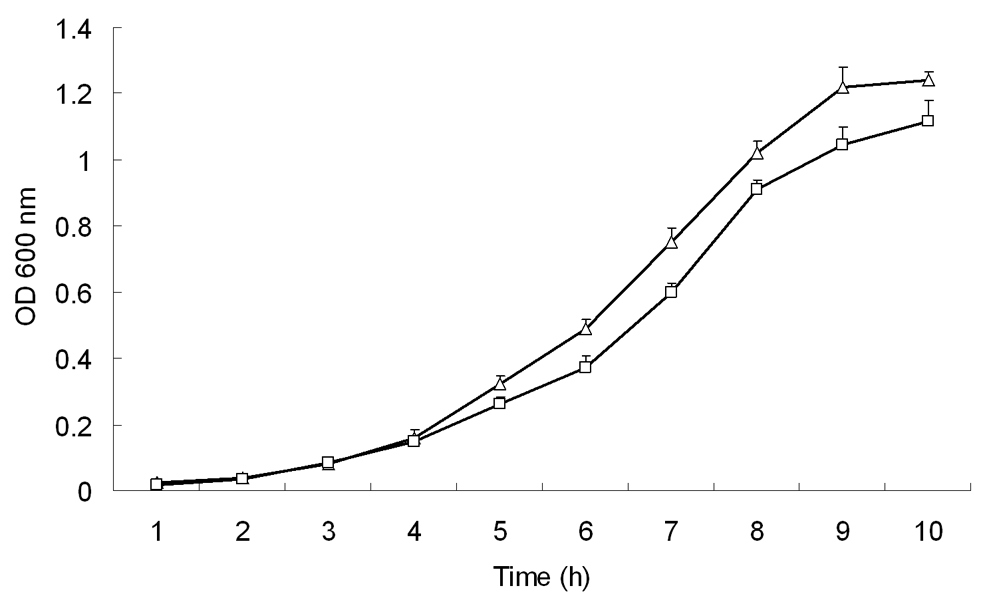

Supplement: Figure S6 — Growth of Δtdh-ΔaaoSo-ΔmutT mutant and the wild-type of S. oligofermentans . Strains were cultured overnight and adjusted to the same OD600 in TYG medium, and 1∶100 diluted with the same medium. OD600 was detected in 1 h interval. Symbols: △, wild-type strain;□, Δtdh-ΔaaoSo-ΔmutT mutant. The results are shown as the means ± SD of three independent experiments. (TIF) [file pone.0038133.s006.tif]
